# Differential RelA- and RelB-dependent gene transcription in LTβR-stimulated mouse embryonic fibroblasts

**DOI:** 10.1186/1471-2164-9-606

**Published:** 2008-12-16

**Authors:** Agnes Lovas, Dörte Radke, Daniela Albrecht, Z Buket Yilmaz, Ulrich Möller, Andreas JR Habenicht, Falk Weih

**Affiliations:** 1 Research Group Immunology, Leibniz Institute for Age Research, Fritz Lipmann Institute, Beutenbergstrasse 11, 07745 Jena, Germany; 2 Bioinformatics – Pattern Recognition, Leibniz Institute for Natural Product Research and Infection Biology, Hans-Knöll-Institute, Beutenbergstrasse 11a, 07745 Jena, Germany; 3 Molecular and Applied Microbiology, Leibniz Institute for Natural Product Research and Infection Biology, Hans-Knöll-Institute, Beutenbergstrasse 11a, 07745 Jena, Germany; 4 Signal Transduction in Tumor Cells, Max Delbrück Center for Molecular Medicine, Robert Rössle Strasse 10, 13092 Berlin-Buch, Germany; 5 Institute for Vascular Medicine, Friedrich Schiller University of Jena, Bachstrasse 18, 07743 Jena, Germany; 6 Institute for Community Medicine, Ernst Moritz Arndt University Greifswald, Walther Rathenau Strasse 48, 17475 Greifswald, Germany

## Abstract

**Background:**

Lymphotoxin signaling via the lymphotoxin-β receptor (LTβR) has been implicated in biological processes ranging from development of secondary lymphoid organs, maintenance of spleen architecture, host defense against pathogens, autoimmunity, and lipid homeostasis. The major transcription factor that is activated by LTβR crosslinking is NF-κB. Two signaling pathways have been described, the classical inhibitor of NF-κB α (IκBα)-regulated and the alternative p100-regulated pathway that result in the activation of p50-RelA and p52-RelB NF-κB heterodimers, respectively.

**Results:**

Using microarray analysis, we investigated the transcriptional response downstream of the LTβR in mouse embryonic fibroblasts (MEFs) and its regulation by the RelA and RelB subunits of NF-κB. We describe novel LTβR-responsive genes that were regulated by RelA and/or RelB. The majority of LTβR-regulated genes required the presence of both RelA and RelB, revealing significant crosstalk between the two NF-κB activation pathways. Gene Ontology (GO) analysis confirmed that LTβR-NF-κB target genes are predominantly involved in the regulation of immune responses. However, other biological processes, such as apoptosis/cell death, cell cycle, angiogenesis, and taxis were also regulated by LTβR signaling. Moreover, LTβR activation inhibited expression of a key adipogenic transcription factor, peroxisome proliferator activated receptor-γ (*pparg*), suggesting that LTβR signaling may interfere with adipogenic differentiation.

**Conclusion:**

Microarray analysis of LTβR-stimulated fibroblasts provided comprehensive insight into the transcriptional response of LTβR signaling and its regulation by the NF-κB family members RelA and RelB.

## Background

NF-κB transcription factors are essential for innate and adaptive immunity, cell survival, cellular stress responses, development and maintenance of lymphoid organ structures, and other biological functions [1-3]. The vertebrate NF-κB family includes five structurally related members, the Rel proteins RelA (p65), RelB, cRel, and the NF-κB proteins p50 and p52. Among the Rel/NF-κB family, only RelA, RelB, and cRel contain C-terminal transcriptional activation domains enabling them to directly regulate transcription. The other two members, p50 and p52, are synthesized as p105 and p100 precursors, respectively. The Rel and NF-κB proteins can form different homo- and heterodimers (for example p50-RelA or p52-RelB) that bind to DNA target sites, so-called κB sites. In resting cells, Rel/NF-κB proteins associate with inhibitory κB molecules (IκBs) and are retained in the cytoplasm as inactive forms [4].

Two major NF-κB signaling pathways can be distinguished, the classical or canonical and the alternative or non-canonical pathway. In response to stimulation of transmembrane receptors like tumor necrosis factor receptor (TNFR)-1 or Toll-like receptor (TLR)-4, signaling cascades are initiated that lead to the liberation of Rel/NF-κB complexes from their IκB molecules. As a result, they translocate to the nucleus and regulate transcription of numerous target genes. This classical pathway involves phosphorylation of IκBα by the NEMO (NF-κB essential modulator)/IKKγ- and IKKβ-containing IκB kinase (IKK) complex followed by its ubiquitin-dependent proteasomal degradation. Regulation of gene transcription is predominantly mediated through p50-RelA and p50-cRel heterodimers and target genes are mainly involved in innate immunity, cell survival, and inflammation. A few inducers of NF-κB, like LTβR, are able to trigger an additional, so-called alternative or non-canonical pathway through the activation of NF-κB-inducing kinase (NIK) and IKKα. The alternative pathway governs gene regulation mainly through p52-RelB heterodimers that are generated from the inactive cytoplasmic p100-RelB complex via signal-dependent processing of the p100 inhibitor to p52. This pathway controls genes that are predominantly involved in adaptive immunity and lymphoid organ development [5-11]. Recent findings by Hoffmann and colleagues extend this scenario. They could show that not only RelB- but also RelA-containing complexes can be released from the p100 inhibitor after LTβR stimulation [12-14].

This report focuses on the transcriptional response downstream of the LTβR and its regulation by RelA and RelB. The role of LTβR signaling in development and organization of secondary lymphoid structures is well documented (reviewed in [8,15-17]). We are interested in similarities and differences in RelA and RelB function in lymphoid organ development. However, a major problem is that RelA-deficient (*relA*^-/-^) mice are embryonic lethal due to tumor necrosis factor (TNF)-induced hepatocyte apoptosis [18]. Moreover, RelB-deficient (*relB*^-/-^) mice display impaired secondary lymphoid organ development and suffer from an autoinflammatory syndrome that also affects organization and function of lymphoid tissues [19,20]. Thus, stromal compartments that display LTβR signaling and thereby have an organizational role in the development of lymphoid organs cannot be used for *in vivo *gene expression studies from the above animals. Therefore, we applied MEFs established from wild-type (wt), *relA*^-/-^, and *relB*^-/- ^mice as an *in vitro *model system. Also, there is increasing evidence that LTβR functions beyond lymphoid organs, as it is involved in liver regeneration, hepatitis [21], and hepatic lipid metabolism [22]. We therefore hypothesized that LTβR signaling, via RelA and/or RelB, may participate in physiological processes other than lymphorganogenesis. MEFs with different genotypes (wt, *relA*^-/-^, and *relB*^-/-^) allowed us to dissect specific RelA and RelB activities in the regulation of gene transcription after LTβR stimulation. In wt MEFs, LTβR signals were predominantly transduced by RelA- and/or RelB-containing dimers. Upon LTβR signaling in *relA*^-/- ^cells, gene regulatory events were mediated by RelB and *vice versa *in *relB*^-/- ^cells, changes in gene expression were mediated by RelA. Using this system, we describe novel LTβR-responsive genes that were regulated solely by RelA or RelB or by both RelA and RelB.

## Results and discussion

### LTβR stimulation of MEFs

**Figure 1:**
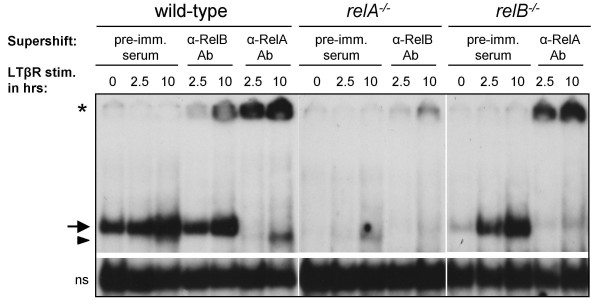
**Confirmation of LTβR stimulation: induction of RelA- and RelB-containing DNA-binding complexes**. Wild-type, *relA*^-/-^, and *relB*^-/- ^MEFs were treated with agonistic anti-LTβR mAb for the indicated times and subsequently nuclear extracts were prepared and analyzed by EMSA for NF-κB DNA-binding activity using an Igκ oligo. Specific Igκ DNA-binding complexes are indicated by arrow (RelA-containing dimers) and arrowhead (RelB-containing dimers). Non-specific DNA binding complexes (ns, lower lane) serve as loading control. Supershift analysis was performed using pre-immune serum (pre-imm. serum), anti-RelA antibody (α-RelA Ab), and anti-RelB antibody (α-RelB Ab). Supershifted complexes are indicated by asterisk.

For LTβR stimulation, MEFs of each genotype were either left untreated or were treated with agonistic anti-LTβR monoclonal antibody (mAb) for 2.5 or 10 h. For each treatment group, cells from four experiments were pooled. Nuclear protein extracts were used in electrophoretic mobility shift assays (EMSAs) to verify proper LTβR signaling (Figure 1). In wt cells, LTβR signaling resulted in modest induction of κB-binding complexes at the early time point (2.5 h) but strong induction after 10 h of stimulation. Dissection of these complexes with supershifting antibodies revealed that the faster migrating complex contained RelB and the slower migrating complex contained RelA. As expected, in wt cells both RelA and RelB complexes were activated in response to LTβR signaling, whereas in *relA*^-/- ^cells only RelB- and in *relB*^-/- ^cells only RelA-containing κB-binding complexes were induced (Figure 1). Recently, slow and relatively weak DNA-binding of NF-κB complexes in response to LTβR ligation was reported [12]. The plateau was reached between 10 and 15 h of LTβR stimulation corresponding to a 2- to 3-fold induction of NF-κB DNA binding. Our results are in agreement with these observations: for each genotype the strongest induction of κB-binding complexes was observed at 10 h. For gene expression profiling we therefore used total RNA isolated from untreated (0 h) and 10 h agonistic anti-LTβR mAb treated wt, *relA*^-/-^, and *relB*^-/- ^MEFs, assuming that stronger DNA-binding activity reflects stronger gene expression changes controlled by NF-κB transcription factor complexes.

### Global gene expression in response to LTβR stimulation in MEFs

**Figure 2:**
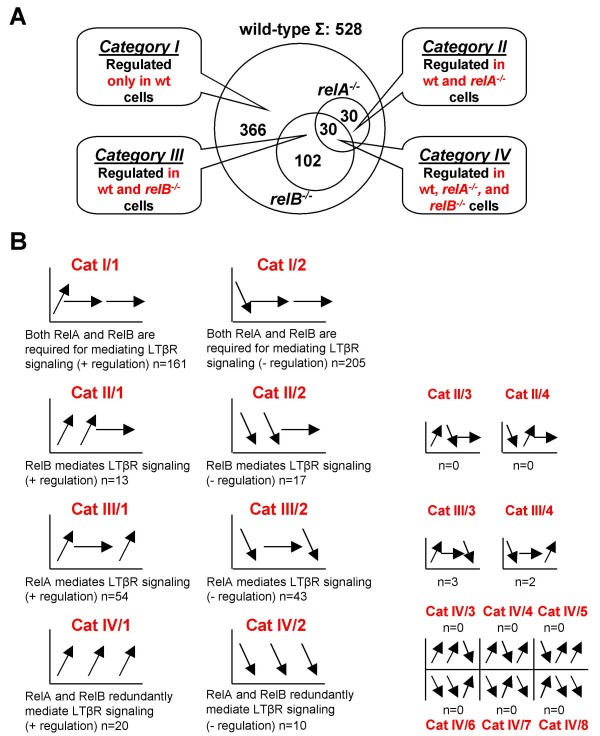
**LTβR-responsive genes can be allocated into distinct categories**. (A) Venn-diagram of significantly (*p *< 0.05) regulated genes. (B) Schematic depiction of gene expression patterns. The four main categories in (A) can be segregated into further subcategories, depending on whether their genes were upregulated or downregulated. The arrows in the plots show the direction of gene expression changes from non-induced (0 h) to the 10 h induced state in response to LTβR stimulation. The first arrow describes gene expression behavior in wild-type, the second in *relA*^-/-^, and the third in *relB*^-/- ^cells. Horizontal arrows indicate lack of change or statistically insignificant change in gene expression. Arrows pointing upwards or downwards indicate significant positive or negative regulation, respectively.

To identify RelA- and RelB-regulated genes after LTβR stimulation, we carried out microarray analysis using total RNA from the experiment described above hybridized to CodeLink UniSet Mouse 20K I bioarrays. For statistical analysis, different genotypes were analyzed separately and significantly differentially expressed genes between time points 0 h and 10 h were identified (*p *< 0.05). The fold change (FC) threshold was determined from the minimal detectable fold change (MDFC) calculated by the CodeLink Expression Analysis v4.1 software (wt: 1.48; *relA*^-/-^: 1.54; *relB*^-/-^: 1.36). In response to LTβR stimulation, a total of 528 genes were regulated in wt cells. In line with the moderate NF-κB activation seen in the EMSAs the observed gene regulation was also modest: gene expression changes were in the range of +5-fold (induction) and -5-fold (repression). We assigned the 528 LTβR-responsive genes to 4 categories: genes that were significantly regulated (i) only in wt cells (category I, n = 366), (ii) in wt and *relA*^-/- ^cells (category II, n = 30), (iii) in wt and *relB*^-/- ^cells (category III, n = 102), and (iv) genes that were significantly regulated in all 3 genotypes (category IV, n = 30) (Figure 2A; for the list of LTβR-responsive genes in wt cells see Additional file 1).

The genes in these four categories could be segregated into further subcategories, which helped us to assign regulatory mechanisms underlying the expression patterns of individual genes (see schematic depiction of gene expression behavior in Figure 2B and lists of genes belonging to different subcategories in Additional files 2, 3, 4, 5).

Category (cat) I genes were significantly regulated only in wt cells in response to LTβR stimulation. This group of genes required both RelA and RelB for their LTβR-dependent activation (cat I/1, n = 161) or repression (cat I/2, n = 205). Therefore, expression of these genes did not significantly change in either of the mutant cell lines in response to agonistic anti-LTβR mAb treatment (Figure 2B, Additional file 2).

Category II genes were significantly regulated in wt and *relA*^-/- ^cells upon LTβR ligation. Genes upregulated (cat II/1, n = 13) or downregulated (cat II/2, n = 17) in both wt and *relA*^-/- ^cells, but not significantly regulated in *relB*^-/- ^cells, were considered to be RelB target genes in response to LTβR signaling. Other theoretical patterns could also be appointed to category II, but we did not find any example in our analysis for these subcategories (cat II/3, n = 0 and cat II/4, n = 0) (Figure 2B, Additional file 3).

Genes belonging to category III were significantly regulated in wt and *relB*^-/- ^cells in response to LTβR stimulation. Genes upregulated (cat III/1, n = 54) or downregulated (cat III/2, n = 43) in both wt and *relB*^-/- ^cells, but not significantly regulated in *relA*^-/- ^cells, were considered to be RelA target genes in response to LTβR signaling. Negligible numbers of genes in category III could also be allocated to cat III/3 and III/4 (n = 3 and n = 2, respectively) (Figure 2B, Additional file 4). However, these genes were not further analyzed. The significantly larger number of RelA- (cat III) compared to RelB-regulated genes (cat II; Figure 2A) is likely to be a consequence of the stronger LTβR-induced DNA binding of RelA compared to RelB complexes (Figure 1).

Category IV genes were significantly regulated in each of the genotypes in response to LTβR ligation. Although eight theoretically possible gene expression behaviors exist, we only found genes that belonged to two easily explainable scenarios: genes were either upregulated (cat IV/1, n = 20), or downregulated (cat IV/2, n = 10) in each genotype upon LTβR signaling (Figure 2B, Additional file 5). Most likely, both RelA and RelB contributed redundantly to their regulation or alternatively, a third factor/pathway controlled these genes in response to LTβR stimulation. JNK (c-Jun N-terminal kinase) is a possible candidate for such a third pathway, as there are indications that LTβR stimulation leads to activation of JNK. However, the experimental setup in those studies was different from ours as LTβR-overexpressing HEK293 cells [23] or treatment of MEFs with the LTβR agonist LIGHT (lymphotoxin-related inducible ligand that competes for glycoprotein D binding to herpesvirus entry mediator on T cells) [24] were studied.

**Figure 3:**
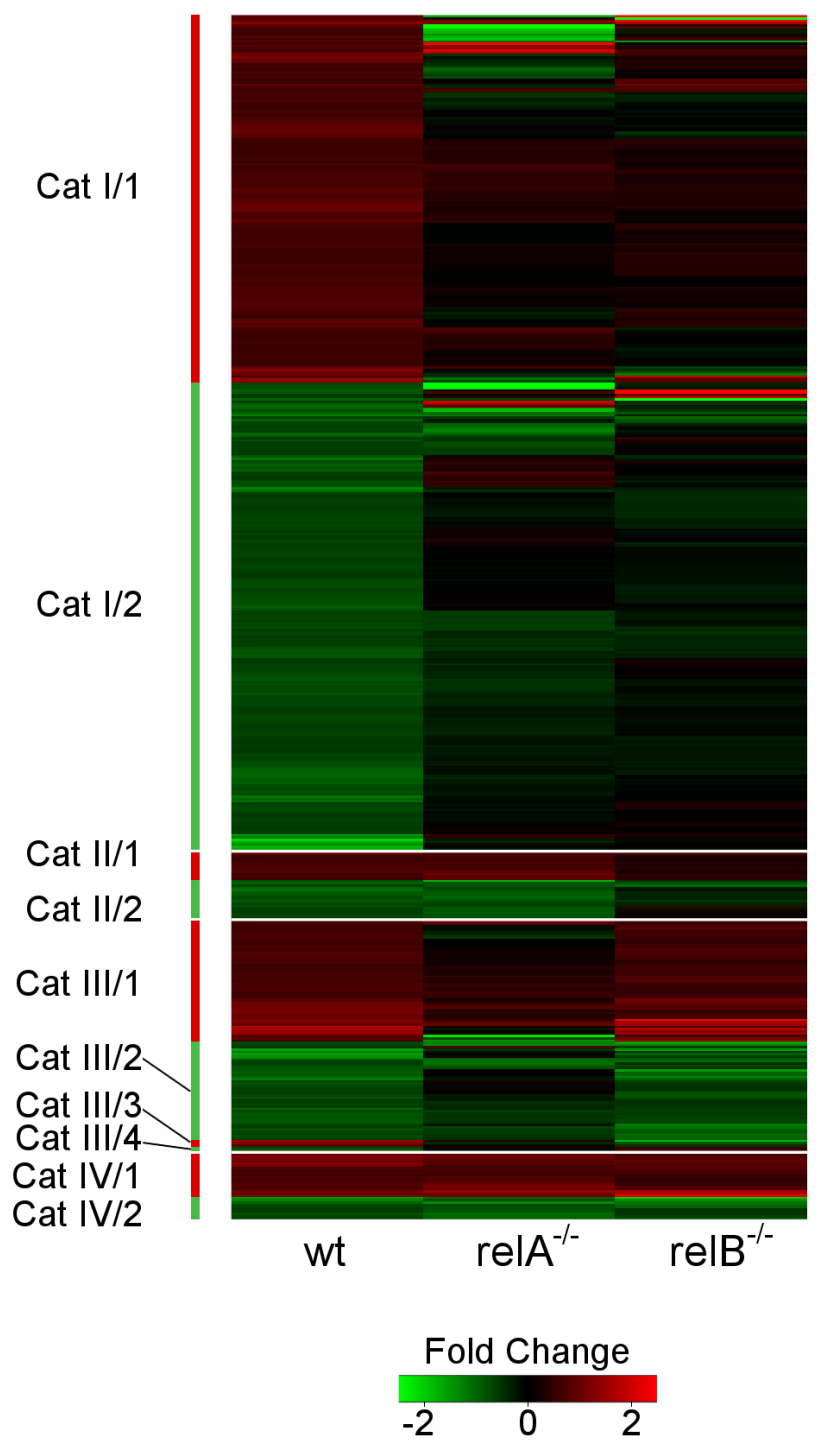
**Fold change heatmaps**. Heatmaps displaying the fold change values observed in the three different cell lines at 10 h compared to 0 h. The color code indicates the fold change values between -2.5-fold downregulation (green) and +2.5-fold upregulation (red). Fold change of -2.5 and below are depicted in the brightest green and fold change of +2.5 and above are shown in the brightest red. Black indicates no change in gene expression. Each horizontal line on the heatmap corresponds to one gene. Genes are arranged by their subcategory (see bars on the left) and main categories are divided by a horizontal white line.

FC values observed in the three cell lines at 10 h compared to 0 h are displayed in a heatmap that also reflects the four categories and their subcategories (Figure 3, for a zoomable/enlarged version of FC heatmaps supplied with gene symbols and GenBank Accession Numbers see Additional file 6).

Interestingly, in the two subcategories with the largest number of genes both RelA and RelB together were required for LTβR-induced gene regulation (161 cat I/1 genes for their activation and 205 cat I/2 genes for their repression). In case one of the transcription factors was missing the other one was not able to ensure regulation alone, suggesting significant crosstalk between the two NF-κB activation pathways. In response to LTβR stimulation, sequential engagement of the classical and alternative pathway was suggested, resulting in initial DNA binding by RelA followed by RelB complexes [7,9]. These findings may suggest a scenario where RelA binds first to the DNA in the promoter of category I genes, loosens up chromatin, thereby enabling subsequent DNA binding and gene regulatory action by RelB [25]. Alternatively, since *relB *is an NF-κB target gene [26] RelA may ensure sufficiently high expression of RelB and in the absence of RelA the reduced RelB levels cannot mediate proper regulation of certain LTβR target genes. This possibility is supported by the observation that in the absence of RelA both RelB protein levels and binding of RelB to κB sites were reduced (Figure 1 and data not shown) [13].

### Meta analysis of LTβR-dependent transcriptomes

LTβR signaling is best known in the context of secondary lymphoid organ development and a recent expression profiling study described LTβR-dependent transcriptomes in lymph nodes and follicular dendritic cells (FDCs) [27]. However, increasing evidence suggests that LTβR also plays a role in non-lymphoid organs such as epithelial tissues during embryonic development [28] and adult liver [21,22].

To interpret our results in the light of other studies investigating LTβR signaling, we compared our LTβR-responsive genes with two recently published LTβR-dependent transcriptomes. Huber *et al*. identified transcripts in murine mesenteric lymph nodes affected *in vivo *by the administration of a soluble LTβR-Ig decoy receptor which blocks LTβR signaling [27]. A gene cluster of 80 unique transcripts that showed decreased expression after LTβR blockade was further analyzed. Twelve genes in this cluster were also associated with germinal centers (GCs)/FDC. A few common genes were found between our analysis and the LTβR-dependent transcriptomes described by Huber *et al*. *Dclk1 *and *enpp2 *(doublecortin-like kinase 1; GenBank Accession Number: NM_019978 and ectonucleotide pyrophosphatase/phosphodiesterase 2 or autotaxin; GenBank Accession Number: NM_015744) expression was moderately decreased 3 d after LTβR blockade (FC: 0.70× and 0.66×, respectively) [27]. In our hands, both genes were upregulated in response to LTβR stimulation in a RelA-dependent manner (cat III/1, for *enpp2 *see also Table 12). *Enpp2 *was also found to be associated with GC/FDC in mesenteric lymph nodes [27]. Moreover, Enpp2 (also called autotaxin) has been recently described as a new molecule in lymphocyte homing through high endothelial venules (HEVs) [29]. Collectively, these findings suggest that LTβR, in addition to its well-described effect on the HEV differentiation program [30], further contributes via RelA-dependent upregulation of *enpp2 *to lymphocyte homing through HEVs. Unfortunately, we could not detect further genes with a similar regulation pattern in our and Huber and colleagues' studies. This lack of overlap could be the consequence of several reasons: (i) different modes of function and kinetics of antagonistic LTβR-Ig vs agonistic anti-LTβR mAb application, (ii) incubation time (3 d treatment with LTβR-Ig vs 10 h treatment with agonistic anti-LTβR mAb), or (iii) *in vivo *collection of different cell types influenced by the treatment vs *in vitro *cell culture system using MEFs.

Lo *et al*. described a hepatic gene expression profile of wt vs lck-LIGHT transgenic mice (overexpressing the LTβR ligand LIGHT on the surface of T lymphocytes) [22]. A group of significantly regulated genes (n = 19) involved in lipid and cholesterol metabolism was identified. The gene that displayed the highest level of regulation (23-fold repression in transgenic vs wt mice) encodes for hepatic lipase, a key enzyme in lipid metabolism. We did not observe repression of hepatic lipase in our experiments, most probably due to its restricted expression on the surface of hepatocytes. However, we found another gene belonging to the lipid/cholesterol metabolism-related group described by Lo and colleagues. *Ralgds *(ral guanine nucleotide dissociation stimulator, GenBank Accession Number: NM_009058) expression was increased in the liver of transgenic mice and also upregulated in our LTβR stimulation experiments, belonging to the RelA-responsive genes (cat III/1, Table 12).

### Gene Ontology (GO) enrichment analysis

Our goal was not only to define the LTβR-dependent transcriptome in MEFs, but also to assign regulatory mechanisms to LTβR signaling, i.e. to examine which part of the LTβR transcriptome is regulated by RelA, RelB, or both. We started out with GO enrichment analysis of significantly regulated genes to identify biological processes, molecular functions, and cellular components putatively regulated in the categories described above. Compared to molecular functions and cellular components, GO analysis of biological processes yielded the most conclusive results.

First, GO analysis was performed on the total LTβR transcriptome in wt cells to see how LTβR signaling influences biological processes in these fibroblasts, regardless whether these genes were also regulated in *relA*^-/- ^or *relB*^-/- ^cells (Category: Total wild-type, Table 1). For interpretation of our data we chose GO terms with *p *< 0.01. As lower limit, we did not consider GO terms with less than 3 annotated genes in the list of differentially regulated genes since they are too specific. As upper limit we did not use GO terms represented by more than 600 genes on the microarray since they are too general. Among the considered GO terms we found that apoptosis/cell death (A/CD)- and cell cycle (CCY)-related processes were overrepresented. We also found that genes annotated with "response to biotic stimulus", "immune system process" (immune related (IR) features) as well as "blood vessel morphogenesis" and "angiogenesis" (blood vessel development related (BR) features) were enriched. Collectively, these data indicate that LTβR signaling largely influences cell survival/cell proliferation features. Moreover, it has an impact on immune responses and blood vessel development/angiogenesis related processes. Since these GO terms were found in LTβR-stimulated "non-immune" fibroblasts it is likely that LTβR signaling regulates similar biological processes in stromal cells of secondary lymphoid tissues governing lymphorganogenesis and maintaining lymphoid tissue architecture.

**Table 1:** Gene Ontology analysis of total LTβR transcriptome in wild-type cells

**GO number**	**GO term**	**Type of biological process**	***p *value**	**n sel**.	**n tot**.
GO:0007049	Cell cycle	CCY	1.80E-05	39	559

GO:0006915	Apoptosis	A/CD	1.00E-04	34	499

GO:0008219	Cell death	A/CD	0.00011	35	523

GO:0016265	Death	A/CD	0.00011	35	523

GO:0012501	Programmed cell death	A/CD	0.00012	34	503

GO:0006259	DNA metabolic process	CCY	0.00016	32	469

GO:0022402	Cell cycle process	CCY	0.00034	30	447

GO:0042981	Regulation of apoptosis	A/CD	0.00063	23	319

GO:0043067	Regulation of programmed cell death	A/CD	0.00068	23	321

GO:0009607	Response to biotic stimulus	IR	0.0035	11	124

GO:0006260	DNA replication	CCY	0.0037	10	107

GO:0043066	Negative regulation of apoptosis	A/CD	0.0045	11	128

GO:0000074	Regulation of progression through cell cycle	CCY	0.0047	19	287

GO:0043069	Negative regulation of programmed cell death	A/CD	0.0048	11	129

GO:0051726	Regulation of cell cycle	CCY	0.0051	19	289

GO:0002376	Immune system process	IR	0.0053	30	534

GO:0030968	Unfolded protein response		0.0054	3	11

GO:0007610	Behavior		0.0054	17	249

GO:0009953	Dorsal/ventral pattern formation		0.0057	5	37

GO:0016567	Protein ubiquitination		0.0064	5	35

GO:0006730	One-carbon compound metabolic process		0.0067	7	65

GO:0048514	Blood vessel morphogenesis	BR	0.0078	12	157

GO:0040029	Regulation of gene expression, epigenetic		0.0082	5	37

GO:0007631	Feeding behavior		0.0084	4	24

GO:0001525	Angiogenesis	BR	0.0087	10	121

GO:0006171	cAMP biosynthetic process		0.0089	3	13

GO:0051094	Positive regulation of developmental process		0.0092	6	53

Analysis of functional enrichment was performed employing Fisher's exact test. The number of genes annotated with a specific GO term was determined for the list of differentially expressed genes (n sel.) and compared to all GO annotated genes on the array (n tot.). The resulting *p *values (*p *< 0.01) were used to rank GO terms according to their significance. Terms with more than 600 genes on the array or less than 3 genes on the list of investigated genes were regarded as too general or too specific, respectively, and excluded from the analysis. A/CD, apoptosis/cell death; CCY, cell cycle; IR, immune related; BR, blood vessel development related.

Next, we carried out GO analysis for the four main categories and for all subcategories with at least 20 genes. Interpretation of the data was performed applying the same criteria as above. GO analysis of category I genes revealed those biological processes that were overrepresented only in LTβR-stimulated wt cells, i.e. in the presence of both RelA and RelB (Table 2). Amongst these processes, CCY-related terms dominated. Subsequently, we analyzed cat I/1 (containing genes that were upregulated exclusively in wt cells) and found enrichment of IR- and cell/biological adhesion (important events in immune cell migration)-related terms on the list of biological processes (Table 3). This finding indicates that in the absence of RelA or RelB a considerable portion of LTβR-stimulated immune response-related events cannot be carried out; fibroblasts need both molecules to execute these processes. In cat I/2 (containing genes that are downregulated exclusively in wt cells) we found enrichment of CCY-related terms on the list of overrepresented biological processes (Table 4). This finding indicates that in wt cells an important action of RelA and RelB is to downregulate numerous genes that are implicated in cell cycle regulation in response to LTβR signaling.

**Table 2:** Gene Ontology analysis of category I

**GO number**	**GO term**	**Type of biological process**	***p *value**	**n sel**.	**n tot**.
GO:0006259	DNA metabolic process	CCY	1.40E-05	27	469

GO:0007049	Cell cycle	CCY	1.80E-05	30	559

GO:0022402	Cell cycle process	CCY	0.00033	23	447

GO:0040029	Regulation of gene expression, epigenetic		0.0016	5	37

GO:0006260	DNA replication	CCY	0.0036	8	107

GO:0022403	Cell cycle phase	CCY	0.0041	12	211

GO:0006730	One-carbon compound metabolic process		0.0041	6	65

GO:0051301	Cell division	CCY	0.0045	11	187

GO:0031497	Chromatin assembly		0.0047	5	47

GO:0016458	Gene silencing		0.0068	3	17

GO:0009953	Dorsal/ventral pattern formation		0.0079	4	34

GO:0043543	Protein amino acid acylation		0.008	3	18

GO:0000278	Mitotic cell cycle	CCY	0.0081	10	175

GO:0016567	Protein ubiquitination		0.0087	4	35

GO analysis was performed the same way as for category "total wild-type" described in Table 1 legend. CCY, cell cycle.

**Table 3:** Gene Ontology analysis of category I/1

**GO number**	**GO term**	**Type of biological process**	***p *value**	**n sel**.	**n tot**.
GO:0045087	Innate immune response	IR	0.0027	4	58

GO:0002526	Acute inflammatory response	IR	0.0037	4	63

GO:0007155	Cell adhesion	IR	0.0054	11	447

GO:0022610	Biological adhesion	IR	0.0054	11	447

GO analysis was performed the same way as for category "total wild-type" described in Table 1 legend. IR, immune related.

**Table 4:** Gene Ontology analysis of category I/2

**GO number**	**GO term**	**Type of biological process**	***p *value**	**n sel**.	**n tot**.
GO:0007049	Cell cycle	CCY	3.10E-07	24	559

GO:0006259	DNA metabolic process	CCY	9.50E-07	21	469

GO:0022402	Cell cycle process	CCY	7.00E-06	19	447

GO:0022403	Cell cycle phase	CCY	2.40E-05	12	211

GO:0051301	Cell division	CCY	3.90E-05	11	187

GO:0000278	Mitotic cell cycle	CCY	0.00011	10	175

GO:0006730	One-carbon compound metabolic process		0.00022	6	65

GO:0006468	Protein amino acid phosphorylation		0.00025	17	487

GO:0006260	DNA replication	CCY	0.00055	7	107

GO:0000279	M phase	CCY	0.00057	9	176

GO:0016310	Phosphorylation		0.00076	17	536

GO:0009953	Dorsal/ventral pattern formation		0.001	4	34

GO:0040029	Regulation of gene expression, epigenetic		0.0014	4	37

GO:0007067	Mitosis	CCY	0.0015	7	126

GO:0000087	M phase of mitotic cell cycle	CCY	0.0015	7	127

GO:0043543	Protein amino acid acylation		0.0016	3	18

GO:0007224	Smoothened signaling pathway		0.0038	3	24

GO:0006913	Nucleocytoplasmic transport		0.004	5	79

GO:0051169	Nuclear transport		0.004	5	79

GO:0007178	Transmembrane receptor protein serine/threonine kinase signaling pathway		0.0083	4	60

GO:0022613	Ribonucleoprotein complex biogenesis and assembly		0.0093	6	135

GO:0035295	Tube development		0.0096	6	136

GO analysis was performed the same way as for category "total wild-type" described in Table 1 legend. CCY, cell cycle.

Since cat II/1 and II/2 had only few genes (n = 13 and n = 17, respectively), investigation of GO terms for these groups of genes was not meaningful. GO analysis of the main category II (containing genes that were regulated – either up or down – in wt and *relA*^-/- ^cells, n = 30) revealed only one enriched GO term, the cell cycle (Table 5). Thus, in response to LTβR signaling a characteristic feature of RelB was to influence cell cycle-related events.

**Table 5:** Gene Ontology analysis of category II

**GO number**	**GO term**	**Type of biological process**	***p *value**	**n sel**.	**n tot**.
GO:0007049	Cell cycle	CCY	0.0059	5	559

GO analysis was performed the same way as for category "total wild-type" described in Table 1 legend. CCY, cell cycle.

Category III contains genes that were regulated – either up or down – in wt and *relB*^-/- ^cells in response to LTβR stimulation. Among enriched biological processes, the new and in previous categories not yet observed theme taxis and response to external/chemical stimulus (T) dominated, but A/CD-related events also appeared (Table 6). As expected, the theme IR was also represented among the enriched biological processes. This shows that RelA is not only a signal transducer for immune responses and apoptosis/cell death, but also has an impact on the transcription of taxis- and stimulus-responsive genes following LTβR ligation. Among the enriched biological processes of cat III/1 we observed again overrepresentation of T and IR processes (Table 7), revealing that in response to LTβR signaling RelA strongly influenced these events via upregulation of several genes. In cat III/2 we found genes that were repressed by RelA. In this subcategory RelA on one hand regulated several BR events. On the other hand, it turned out to be a negative regulator of genes involved in ion homeostasis (ION) downstream of the LTβR (Table 8).

**Table 6:** Gene Ontology analysis of category III

**GO number**	**GO term**	**Type of biological process**	***p *value**	**n sel**.	**n tot**.
GO:0006939	Smooth muscle contraction		0.00018	3	16

GO:0048675	Axon extension		0.00027	3	18

GO:0006935	Chemotaxis	T	0.00058	5	95

GO:0042330	Taxis	T	0.00058	5	95

GO:0009605	Response to external stimulus	T	0.0011	9	364

GO:0006936	Muscle contraction		0.0011	4	64

GO:0007610	Behavior	T	0.002	7	249

GO:0048858	Cell projection morphogenesis		0.003	6	200

GO:0032990	Cell part morphogenesis		0.003	6	200

GO:0030030	Cell projection organization and biogenesis		0.003	6	200

GO:0007626	Locomotory behavior	T	0.0072	5	169

GO:0042981	Regulation of apoptosis	A/CD	0.0077	7	319

GO:0043067	Regulation of programmed cell death	A/CD	0.0079	7	321

GO:0042221	Response to chemical stimulus	T	0.0082	7	323

GO:0006915	Apoptosis	A/CD	0.009	9	499

GO:0012501	Programmed cell death	A/CD	0.0094	9	503

GO:0048522	Positive regulation of cellular process		0.0096	10	596

GO:0006955	Immune response	IR	0.0097	7	334

GO analysis was performed the same way as for category "total wild-type" described in Table 1 legend. T, taxis, response to external/chemical stimulus; A/CD, apoptosis/cell death; IR, immune related.

**Table 7:** Gene Ontology analysis of category III/1

**GO number**	**GO term**	**Type of biological process**	***p *value**	**n sel**.	**n tot**.
GO:0006955	Immune response	IR	2.00E-04	7	334

GO:0009605	Response to external stimulus	T	0.00034	7	364

GO:0006935	Chemotaxis	T	0.00041	4	95

GO:0042330	Taxis	T	0.00041	4	95

GO:0002376	Immune system process	IR	0.00065	8	534

GO:0007610	Behavior	T	0.0022	5	249

GO:0007626	Locomotory behavior	T	0.0035	4	169

GO:0006954	Inflammatory response	IR	0.0036	4	171

GO:0006952	Defense response	IR	0.0052	5	305

GO:0002252	Immune effector process	IR	0.0064	3	102

GO:0042221	Response to chemical stimulus	T	0.0066	5	323

GO analysis was performed the same way as for category "total wild-type" described in Table 1 legend. IR, immune related; T, taxis, response to external/chemical stimulus.

**Table 8:** Gene Ontology analysis of category III/2

**GO number**	**GO term**	**Type of biological process**	***p *value**	**n sel**.	**n tot**.
GO:0006939	Smooth muscle contraction		1.40E-05	3	16

GO:0006936	Muscle contraction		3.90E-05	4	64

GO:0001525	Angiogenesis	BR	0.00046	4	121

GO:0048514	Blood vessel morphogenesis	BR	0.0012	4	157

GO:0048646	Anatomical structure formation	BR	0.0012	4	159

GO:0030005	Cellular di-, tri-valent inorganic cation homeostasis	ION	0.0016	3	77

GO:0055066	Di-, tri-valent inorganic cation homeostasis	ION	0.0017	3	78

GO:0008015	Circulation	BR	0.0017	3	79

GO:0030003	Cellular cation homeostasis	ION	0.0021	3	84

GO:0001568	Blood vessel development	BR	0.0021	4	182

GO:0055080	Cation homeostasis	ION	0.0021	3	85

GO:0006873	Cellular ion homeostasis	ION	0.0022	3	86

GO:0055082	Cellular chemical homeostasis	ION	0.0022	3	86

GO:0001944	Vasculature development	BR	0.0023	4	185

GO:0050801	Ion homeostasis	ION	0.003	3	96

GO:0065008	Regulation of biological quality		0.004	5	354

GO:0065008	Chemical homeostasis	ION	0.0062	3	124

GO:0007507	Heart development	BR	0.0088	3	141

GO analysis was performed the same way as for category "total wild-type" described in Table 1 legend. BR, blood vessel development related; ION, ion homeostasis.

Category IV contains genes that were regulated – either up or down – in each of the cell types in response to LTβR stimulation (Table 9). IR processes were overrepresented, but the terms related to hematopoietic or lymphoid organ development (LY) and taxis (T) were also present on the list of enriched biological processes. Unfortunately, we could not analyze cat IV/2, as it comprises too few genes (n = 10). Cat IV/1 contains 20 genes that were upregulated, irrespective of the genotype (Table 10). These genes primarily belong to IR and T. Possibly, RelA and RelB redundantly regulate these events or alternatively a RelA- and RelB-independent third factor/pathway (e.g. JNK) controls these biological processes following LTβR ligation. Table 11 shows a summary of our GO analysis.

**Table 9:** Gene Ontology analysis of category IV

**GO number**	**GO term**	**Type of biological process**	***p *value**	**n sel**.	**n tot**.
GO:0002376	Immune system process	IR	4.40E-05	7	534

GO:0006955	Immune response	IR	0.00038	5	334

GO:0045595	Regulation of cell differentiation		0.0013	3	113

GO:0006952	Defense response	IR	0.0026	4	305

GO:0042221	Response to chemical stimulus	T	0.0032	4	323

GO:0006954	Inflammatory response	IR	0.0043	3	171

GO:0048534	Hemopoietic or lymphoid organ development	LY	0.0064	3	197

GO:0050793	Regulation of developmental process		0.0067	3	201

GO:0002520	Immune system development	IR	0.0078	3	212

GO analysis was performed the same way as for category "total wild-type" described in Table 1 legend. IR, immune related; T, taxis, response to external/chemical stimulus; LY, hematopoietic or lymphoid organ developmental processes.

**Table 10:** Gene Ontology analysis of category IV/1

**GO number**	**GO term**	**Type of biological process**	***p *value**	**n sel**.	**n tot**.
GO:0002376	Immune system process	IR	1.40E-05	6	534

GO:0006955	Immune response	IR	2.4E-05	5	334

GO:0006952	Defense response	IR	0.00032	4	305

GO:0006954	Inflammatory response	IR	0.00091	3	171

GO:0009611	Response to wounding	IR	0.0024	3	240

GO:0015031	Protein transport		0.0024	4	523

GO:0045184	Establishment of protein localization		0.0029	4	546

GO:0008104	Protein localization		0.0037	4	586

GO:0042221	Response to chemical stimulus	T	0.0056	3	323

GO:0006886	Intracellular protein transport		0.0057	3	326

GO:0009605	Response to external stimulus	T	0.0078	3	364

GO analysis was performed the same way as for category "total wild-type" described in Table 1 legend. IR, immune related; T, taxis, response to external/chemical stimulus.

**Table 11:** Summary of Gene Ontology analysis results

**Category/Subcategory**	**Enriched biological processes**	**Regulatory molecules downstream of LTβR, and their effects on the gene expression**
Total wild-type	A/CD CCY IR BR	Molecules not assignable – up and downregulation

Cat I	CCY	RelA and RelB together – up and downregulation

Cat I/1	IR	RelA and RelB together – upregulation

Cat I/2	CCY	RelA and RelB together – downregulation

Cat II	CCY	RelB – up and downregulation

Cat II/1	Not investigated	RelB – upregulation

Cat II/2	Not investigated	RelB – downregulation

Cat III	T A/CD IR	RelA – up and downregulation

Cat III/1	T IR	RelA – upregulation

Cat III/2	ION BR	RelA – downregulation

Cat IV	IR T LY	RelA and RelB via redundant effects – up and downregulation **OR** Third pathway – up and downregulation

Cat IV/1	IR T	RelA and RelB via redundant effects – upregulation **OR** Third pathway – upregulation

Cat IV/2	Not investigated	RelA and RelB via redundant effects – downregulation **OR** Third pathway – downregulation

Summary of GO analysis: categories/subcategories with their respective enriched biological processes and the assigned regulatory mechanisms are listed. A/CD, apoptosis/cell death; CCY, cell cycle; IR, immune related; BR, blood vessel development related; T, taxis, response to external/chemical stimulus; ION, ion homeostasis; LY, hematopoietic or lymphoid organ developmental processes. Since cat II/1, II/2 and cat IV/2 had only few genes (n = 13, 17 and 10, respectively) they were not investigated for GO terms.

### Verification of microarray results by qRT-PCR

The changes in mRNA levels of several known as well as novel LTβR-responsive genes on the microarray were confirmed by quantitative real-time reverse-transcription-PCR (qRT-PCR), using RNA from three independent LTβR stimulation experiments (Table 12). In agreement with previous reports, we also found induction of *nfkb2 *[5,6], *ccl2/mcp1 *[6], and *ikba *expression [31] in LTβR-stimulated wt fibroblasts. In addition, our data indicate that both RelA and RelB redundantly contributed to the proper regulation of these genes in response to LTβR stimulation. However, we did not observe LTβR-dependent upregulation of lymphorganogenic chemokines as described by others. *Ccl21*, *ccl19*, *cxcl13*, and *cxcl12 *were shown to be LTβR-induced genes in spleen 8 h after peritoneal injection of an agonistic anti-LTβR mAb [5]. Possibly, cell context-specific signaling accounts for the difference observed between splenocytes and established 3T3 fibroblasts used in our experiments. Basak *et al*. observed modest upregulation of *cxcl13 *and *ccl21 *in established wt 3T3 fibroblasts after 24 h treatment with agonistic anti-LTβR mAb [13]. To reduce indirect gene regulatory effects due to rather long stimulation we activated LTβR signaling only for 10 h, where modulation of these chemokines was not observed.

**Table 12:** Verification of microarray results by qRT-PCR

**Gene Symbol and GenBank Accession Number**	**CodeLink bioarrays FC and *p *value (in brackets) for wt/*relA*^-/-^/*relB*^-/- ^cells and corresponding subcategory**	**qRT-PCR FC ± SD for wt/*relA*^-/-^/*relB*^-/- ^cells and corresponding subcategory**
**Cx3cl1** NM_009142	1.77 (0.00370)/0.90 (>0.05)/0.96 (>0.05), **I/1**	1.66 ± 0.22/0.89 ± 0.10/1.08 ± 0.29, **I/1**

**Pparg** NM_011146	0.65 (0.00690)/0.55 (0.01800)/1.32 (>0.05), **II/2**	0.50 ± 0.02/0.48 ± 0.04/0.81 ± 0.11, **II/2**

Ralgds * NM_009058	2.24 (0.00750)/1.48 (>0.05)/1.58 (0.00140), **III/1**	2.03 ± 0.42/1.13 ± 0.10/1.17 ± 0.16, **I/1 – not verified in *relB*^-/- ^cells**

Enpp2 * NM_015744	2.28 (0.00150)/1.36 (>0.05)/5.10 (0.00070), **III/1**	1.85 ± 0.30/1.35 ± 0.27/3.29 ± 0.91, **III/1**

Birc3 NM_007464	2.77 (0.00090)/1.34 (>0.05)/2.94 (0.00140), **III/1**	2.86 ± 0.73/1.27 ± 0.11/2.99 ± 0.47, **III/1**

**Cxcl10/IP10** NM_021274	1.91 (0.00450)/0.58 (>0.05)/2.14 (0.03000), **III/1**	2.58 ± 0.21/1.28 ± 0.39/2.67 ± 0.20, **III/1**

Irf1 NM_008390	1.96 (0.00270)/2.05 (>0.05)/2.90 (0.00075), **III/1**	2.67 ± 0.32/1.77 ± 0.77/2.15 ± 0.19, **III/1**

**Cd74** NM_010545	3.11 (0.00300)/0.82 (>0.05)/3.46 (0.00070), **III/1**	5.01 ± 0.99/1.06 ± 0.18/4.77 ± 0.56, **III/1**

Fosl1 NM_010235	0.49 (0.00290)/0.86 (>0.05)/0.42 (0.00070), **III/2**	0.46 ± 0.09/0.90 ± 0.09/0.47 ± 0.10, **III/2**

**Nfkb2** NM_019408	2.18 (0.0029)/1.57 (0.0016)/1.81 (0.0007), **IV/1**	2.04 ± 0.37/2.43 ± 0.50/2.74 ± 0.54, **IV/1**

**Ccl2/MCP1** NM_011333	2.10 (0.00120)/2.84 (0.0011)/2.99 (0.00099), **IV/1**	2.29 ± 0.42/3.18 ± 0.13/6.31 ± 1.63, **IV/1**

**Nfkbia/IκBα** NM_010907	2.00 (0.00064)/2.19 (0.00270)/3.42 (0.00140), **IV/1**	1.77 ± 0.16/2.44 ± 0.34/3.92 ± 0.42, **IV/1**

**Ccl7/MCP3** NM_013654	2.22 (0.00041)/1.99 (0.04700)/4.35 (0.00140), **IV/1**	2.77 ± 0.13/3.15 ± 0.15/5.29 ± 1.68, **IV/1**

Cxcl1/KC NM_008176	2.40 (0.00580)/1.77 (0.01600)/1.80 (0.00160), **IV/1**	2.40 ± 0.46/1.31 ± 0.61/3.41 ± 0.88, **III/1 – not verified in *relA*^-/- ^cells**

Id2 NM_010496	0.42 (0.00440)/0.60 (0.04000)/0.39 (0.00075), **IV/2**	0.47 ± 0.11/0.75 ± 0.05/0.57 ± 0.14, **IV/2**

qRT-PCR using RNA from 3 independent LTβR stimulation experiments confirmed changes in mRNA levels of several known as well as novel LTβR-responsive genes on the microarray. Gene names (Gene Symbol) and GenBank Accesion Numbers are shown in the first column. FC values with corresponding *p *values in brackets, observed in the 3 cell lines (wt; *relA*^-/-^; *relB*^-/-^) at 10 h with CodeLink bioarrays and corresponding subcategories (in bold) are displayed in the second column. FC values with corresponding standard deviations (SD), observed in the 3 cell lines (wt; *relA*^-/-^; *relB*^-/-^) at 10 h with qRT-PCR using RNA from 3 independent LTβR stimulation experiments and corresponding subcategories (in bold) are displayed in the third column. Genes that are discussed in chapter "Meta analysis of LTβR-dependent transcriptomes" are indicated by an asterisk and genes that are discussed in chapter "Verification of microarray results by qRT-PCR" are listed in bold.

**Table 13:** LTβR responsive qRT-PCR verified genes in literature

**Gene Symbol and GenBank Accession Number**	**LTβR responsiveness** „reference“ if known/„this study“ if new	**In response to LTβR stimulation, transcription is regulated by RelA or RelB, + or - or 0 manner **„reference“ if known/„this study“ if new
**Cx3cl1** NM_009142	This study	+ regulation by RelA and RelB **together**, this study

**Pparg** NM_011146	This study	0 RelA, this study - RelB, this study

Ralgds * NM_009058	Lo *et al*., 2007 [22]	Mode of regulation uncertain: RelA either alone, or together with RelB enhances Ralgds expression.

Enpp2 * NM_015744	Huber *et al*., 2005 [27]	+ RelA, this study 0 RelB, this study

Birc3 NM_007464	This study	+ RelA, this study 0 RelB, this study

**Cxcl10/IP10** NM_021274	Lukashev *et al*., 2006 [34]	+ RelA, this study 0 RelB, this study

Irf1 NM_008390	Kutsch *et al*., 2008 [41]	+ RelA, this study 0 RelB, this study

**Cd74** NM_010545	This study	+ RelA, this study 0 RelB, this study

Fosl1 NM_010235	This study	- RelA, this study 0 RelB, this study

**Nfkb2** NM_019408.1	Dejardin *et al*., 2002 [5] Derudder *et al*., 2003 [6]	+ RelA, Dejardin *et al*., 2002 [5] + RelB, this study

**Ccl2/MCP1** NM_011333	Derudder *et al*., 2003 [6]	+ RelA, this study + RelB, this study

**Nfkbia/IκBα** NM_010907	Bonizzi *et al*., 2004 [31]	+ RelA, this study + RelB, this study

**Ccl7/MCP3** NM_013654	This study	+ RelA, this study + RelB, this study

Cxcl1/KC NM_008176	This study	+ RelA, this study Positive regulation by RelB is uncertain.

Id2 NM_010496	This study	- RelA, this study - RelB, this study

Genes that are discussed in chapter "Meta analysis of LTβR-dependent transcriptomes" are indicated by an asterisk and genes that are discussed in chapter "Verification of microarray results by qRT-PCR" are listed in bold.

Importantly, we verified novel LTβR-responsive genes and appointed regulatory molecules to them. For a complete list of verified genes see Table 12. Here, some of those verified genes are discussed in more detail.

GO analysis revealed that LTβR stimulation resulted in the regulation of IR processes (Table 11). Except category "Total wild-type", where we could not assign regulatory molecules, in all categories where IR processes were enriched, RelA alone or together with RelB acted as a positive factor. *Cx3cl1 *(chemokine C-X3-C motif ligand 1/fractalkine) is one of the IR genes in cat I/1. Several studies document that NF-κB upregulates *cx3cl1*, e.g. in rat aortic endothelial cells upon interleukin-1β (IL-1β), TNF, and lipopolysaccharide treatment [32] or in human coronary artery smooth muscle cells [33]. The latter work shows that atherogenic lipids induce adhesion of artery smooth muscle cells to macrophages via the upregulation of *cx3cl1 *in a TNF/NF-κB-dependent manner. In our experiments this gene was upregulated in response to LTβR stimulation dependent on RelA and RelB. This data suggests that LTβR, via employing RelA and RelB together, may act as a proatherogenic factor.

IR- and T-related processes were also enriched in cat III and cat III/1 according to the GO analysis. *Cd74/ii *(invariant polypeptide of major histocompatibility complex, class II antigen-associated) and *cxcl10/ip10 *(chemokine C-X-C motif ligand 10/interferon-inducible protein-10) are two genes in cat III/1 and assigned to IR and T. CD74/Ii is involved in antigen processing and presentation and CXCL10 is chemotactic for monocytes and T cells. Moreover, expression of CXCL10, along with two other CXCR3-binding chemokines CXCL9 and CXCL11, can be induced in carcinoma cells by LTβR agonists. These chemokines function as potent chemoattractants for activated T, NK, and dendritic cells, which may contribute to antitumor immune responses [34]. In our experiments, expression of *cd74/ii *and *cxcl10/ip10 *was upregulated by LTβR signaling in wt and *relB*^-/- ^cells. Thus, LTβR signaling via RelA may (i) attract T lymphocytes and promote antigen presentation by dendritic cells in the context of MHC class II and (ii) facilitate antitumor responses against cancer cells.

As indicated by GO analysis, IR- and T-related biological processes were significantly regulated in cat IV and cat IV/1. Amongst others, genes encoding proteins that participate in innate immune responses, like *ccl7/mcp3*, are also represented in these groups. *Ccl7/mcp3 *encodes the proinflammatory chemokine C-C motif ligand 7/monocyte chemotactic protein-3. Expression of *ccl7/mcp3 *was upregulated by LTβR signaling in each of the genotypes, indicating redundant positive regulation by RelA and RelB or upregulation via another RelA- and RelB-independent pathway.

Collectively, positive regulation of the expression of proinflammatory chemokines like *cx3cl1*, *cxcl10*, *ccl7 *(but also others, see Table 12) by LTβR suggests that LTβR signaling, besides regulating development and organization of secondary lymphoid structures, also participates in innate/inflammatory immune responses and for that primarily RelA action seems to be necessary.

Moreover, we found that LTβR signaling functions beyond the regulation of immune responses and organization of lymphoid structures. PPARγ (peroxisome proliferator activated receptor γ) is a key-regulatory transcription factor in the process of adipocyte differentiation and activation of PPARγ promotes the storage of fat [35]. The work of Fu and colleagues suggests that LTβR affects lipid homeostasis by downregulating hepatic lipase expression [22]. Hepatic lipase is expressed on the surface of hepatocytes in the liver. It promotes receptor-mediated uptake of plasma lipoproteins that harbor triglycerides and cholesterol and specifically catalyzes hydrolysis of triglycerides, actions that are suppressed when LTβR signaling is switched on. Expression of *pparg *was negatively affected by LTβR signaling in wt and *relA*^-/- ^but not in *relB*^-/- ^cells (belonging to cat II/2 genes), indicating that this gene was downregulated by RelB in response to LTβR stimulation. Our finding is a further indication that LTβR signaling represses lipogenesis and it may do so via RelB. It has been shown that ligand-induced transactivation by PPARγ is suppressed by IL-1 and TNF and that this suppression is mediated through NF-κB (p50-RelA) [36]. However, unlike suppression of PPARγ by p50-RelA, where this heterodimer blocks PPARγ binding to DNA by forming a complex with PPARγ and its co-activator PGC-2, LTβR-mediated suppression of *pparg *occurred via transcriptional repression executed by RelB. Further experiments are required to find out whether RelB directly or indirectly mediates repression of *pparg *transcription in response to LTβR signaling. The repressive effect of LTβR signaling on adipogenesis has been confirmed in MEFs that were induced for adipogenic differentiation. LTβR stimulation resulted in attenuated lipid droplet accumulation as well as in reduced *pparg *and adipogenic marker gene (*fabp4/ap2*) expression under conditions that promote differentiation into adipocytes (unpublished results).

## Conclusion

This study is the first systematic dissection of the RelA- and RelB-driven transcriptome response downstream of the LTβR. We confirmed previously described LTβR-regulated genes. More importantly, we identified novel LTβR-responsive genes and assigned underlying regulatory mechanisms executed by RelA and/or RelB to them (Table 13). We found that the majority of LTβR-regulated genes required the presence of both RelA and RelB, suggesting significant crosstalk between the two NF-κB activation pathways. Gene Ontology analysis confirmed that LTβR-NF-κB target genes were predominantly involved in the regulation of immune responses. However, other biological processes such as apoptosis/cell death, cell cycle, angiogenesis, and taxis were also regulated by LTβR signaling. Furthermore, we show that LTβR stimulation downregulated expression of the gene encoding PPARγ, suggesting that LTβR signaling may repress adipogenic differentiation by attenuating the levels of this key adipogenic transcription factor. Our findings are significant since they indicate a role for LTβR signaling beyond immune responses and lymphoid organ development and assign underlying gene expression regulatory mechanisms to the LTβR transcriptome.

## Methods

### Cell culture

Mouse embryonic 3T3 fibroblasts (wild-type, *relA*^-/-^, and *relB*^-/-^; kind gift from A. Hoffmann) were cultured at 37°C in Dulbecco's modified Eagle's medium (GIBCO/Invitrogen, Karlsruhe, Germany) supplemented with 10% heat-inactivated bovine calf serum (Perbio Science, Bonn, Germany), penicillin (100 U/ml), streptomycin (100 μg/ml), and Glutamax I (2 mM) (GIBCO/Invitrogen) and treated with agonistic anti-LTβR mAb (1 μg/ml, clone AC.H6; kind gift from J. Browning and P. Rennert).

### EMSA

Preparation of nuclear extracts and EMSAs were essentially performed as previously described [37]. Nuclear and cytoplasmic fractions were prepared according to standard procedures [38].

### RNA isolation

Total cellular RNA was isolated using the RNeasy Mini Kit (Qiagen, Hilden, Germany) according to the manufacturer's instructions. Possible contamination by genomic DNA was removed by DNaseI treatment using the RNase-Free DNase Set (Qiagen). Quality of RNA samples was checked by spectrophotometry and agarose gel electrophoresis. RNAs (2 μg total RNA per sample) were used for cRNA preparation for microarrays only when the ratio A260:A280 was 1.8–2.1 and the RNA was intact.

### Microarrays

Microarray analysis was performed using CodeLink UniSet Mouse 20K I bioarrays (GE Healthcare, Munich, Germany), a one-color system where for each of the investigated 19,801 transcripts there is one 30–mer oligo probe spotted per slide. For gene expression profiling, untreated (0 h) and 10 h agonistic anti-LTβR mAb treated wt, *relA*^-/-^, and *relB*^-/- ^MEFs were used. For every treatment group, cells from 4 experiments were pooled, total RNA isolated, cRNA prepared and hybridized onto the bioarrays in technical triplicates. cRNA target preparation, bioarray hybridization and detection were carried out according to the manufacturer's protocol provided with the CodeLink Expression Assay Reagent Kit. For scanning microarrays, a GenePix 4000B Array Scanner and GenePix Pro 4.0 software (Axon Instruments Inc./Molecular Devices, Munich, Germany) were employed according to settings suggested by the protocol provided with the CodeLink Expression Assay Reagent Kit. Microarray data have been deposited in NCBIs GEO  and are accessible through GEO series accession number GSE11963.

### Microarray data preprocessing

Microarray raw data of stimulated and unstimulated MEFs were analyzed using the Codelink™ Expression Analysis v4.1 software (GE Healthcare) and MDFC values were extracted. All subsequent analyses were performed using R and Bioconductor. For the analysis only genes with probe type 'DISCOVERY' were considered (19,801 genes) and all genes flagged MSR (Manufactory Slide Report) in any sample were excluded (leaving 19,580 genes). To remove negative expression values (local background > spot intensity) raw intensities with values < 0.01 were set to 0.01. The raw intensities of each array were scaled to the array median. After logarithmizing the expression values quantile normalization was applied across all arrays.

### Differentially expressed genes

Array data for the different genotypes were analyzed separately. A gene was included in the analysis if it was flagged 'G' (good) or 'S' (contains saturated pixels) on at least two arrays in any of the two groups (stimulated or unstimulated). Furthermore, genes selected were required to have a FC higher than or equal to the FC threshold determined from the maximum MDFC in these groups. To identify genes significantly differentially expressed after stimulation, a Student's *t*-test was performed for the previously filtered genes. The resulting *p *values were corrected for multiple testing using the method of Benjamini and Hochberg [39]. Allowing a false discovery rate of 5%, a total of 528 genes were identified that were significantly regulated in wt cells (regardless whether they were regulated somewhere else). From these, 366 genes were regulated exclusively in wt, 30 genes in wt and *relA*^-/-^, 102 in wt and *relB*^-/- ^cells and 30 genes in all 3 genotypes.

### Functional analysis with GO

Analysis of functional enrichment was performed employing Fisher's exact test. The resulting *p *values (*p *< 0.01) were used to rank GO terms according to their significance. Terms with more than 600 genes on the array or less than 3 genes on the list of investigated genes were regarded as too general or too specific, respectively, and excluded from the analysis. Expert knowledge was used to assign broader themes to specific GO categories.

### qRT-PCR

For qRT-PCR, first strand cDNA was obtained from 2 μg of total RNA for each treatment group using oligo-dT primers and M-MLV Reverse Transcriptase kit (Promega, Mannheim, Germany) according to manufacturer's protocols. qRT-PCRs were performed in an iCycler Thermal Cycler real-time PCR machine (Bio-Rad Laboratories, Hercules, CA) using SYBR Green I as detector dye and reagents from the Quantace SensiMix DNA Kit (Quantace Ltd., Watford, UK). Primers for qRT-PCRs with Tm of 60°C were designed using Primer3 software (v. 0.4.0; ) [40]. For individual samples, each gene was tested in triplicates and the mean of the 3 cycle threshold values was used to calculate relative expression levels. For normalization, *β-actin *was used as an endogenous reference gene to correct for variation in RNA content and variation in the efficiency of the reverse transcription reaction. Statistical analysis of qRT-PCR results from 3 independent LTβR stimulation experiments was performed employing a Welch test. Forward (F) and reverse primers (R) in 5' to 3' orientation were: Nfkb2_F: GCTAATGTGAATGCCCGGAC, Nfkb2_R: CTTTGGGTATCCCTCTCAGGC, Ccl2_F: CCCACTCACCTGCTGCTACT, Ccl2_R: TCTGGACCCATTCCTTCTTG, IκBα_F: TGCACTTGGCAATCATCCAC, IκBα_R: TTCCTCGAAAGTCTCGGAGCT, Ralgds_F: CATCCACCGCCTAAAGAAGA, Ralgds_R: GGGCTCTCCTAGGGTTCATC, Cx3cl1_F: GGCTAAGCCTCAGAGCATTG, Cx3cl1_R: CATTTTCCTCTGGGGTTGA, Pparg_F: TCATGACCAGGGAGTTCCTC, Pparg_R: GGCGGTCTCCACTGAGAATA, Enpp2_F: TGGCTTACGTGACATTGAGG, Enpp2_R: GTCGGTGAGGAAGGATGAAA, Birc3_F: TGACGTGTGTGACACCAATG, Birc3_R: TGAGGTTGCTGCAGTGTTTC, Cxcl10_F: AAGTGCTGCCGTCATTTTCT, Cxcl10_R: GTGGCAATGATCTCAACACG, Irf1_F: ACCCTGGCTAGAGATGCAGA, Irf1_R: TTTGTATCGGCCTGTGTGAA, Cd74_F: ATGACCCAGGACCATGTGAT, Cd74_R: CCAGGGAGTTCTTGCTCATC, Fosl1_F: CAAAATCCCAGAAGGAGACAAG, Fosl1_R: AAAAGGAGTCAGAGAGGGTGTG, Ccl7_F: AATGCATCCACATGCTGCTA, Ccl7_R: ATAGCCTCCTCGACCCACTT, Cxcl1_F: GCTGGGATTCACCTCAAGAA, Cxcl1_R: TGGGGACACCTTTTAGCATC, Id2_F: CCCCAGAACAAGAAGGTGAC, Id2_R: ATTCAGATGCCTGCAAGGAC, β-actin_F: TGGCGCTTTTGACTCAGGA, β-actin_R: GGGAGGGTGAGGGACTTCC

## Abbreviations

LTβR: lymphotoxin-β receptor; IκBα: inhibitor of NF-κB α; MEF: mouse embryonic fibroblasts; GO: Gene Ontology; PPARγ/*pparg*: peroxisome proliferator activated receptor-γ; TNFR1: tumor necrosis factor receptor 1; TLR4: Toll-like receptor 4; NEMO: NF-κB essential modulator; IKK: IκB kinase; NIK: NF-κB-inducing kinase; *relA*^-/-^: RelA-deficient; TNF: tumor necrosis factor; *relB*^-/-^: RelB-deficient; wt: wild-type; mAb: monoclonal antibody; EMSA: electrophoretic mobility shift assay; FC: fold change; MDFC: minimal detectable fold change; cat: category; JNK: c-Jun N-terminal kinase; LIGHT: lymphotoxin-related inducible ligand that competes for glycoprotein D binding to herpesvirus entry mediator on T cells; FDC: follicular dendritic cell; GC: germinal center; HEV: high endothelial venule; A/CD: apoptosis/cell death; CCY: cell cycle; IR: immune related; BR: blood vessel development related; T: taxis, response to external/chemical stimulus; ION: ion homeostasis; LY: hematopoietic or lymphoid organ developmental processes; qRT-PCR: quantitative real-time reverse-transcription PCR; IL-1: interleukin-1; MSR: Manufactory Slide Report; SD: standard deviation.

## Authors' contributions

AL: carried out the molecular genetic studies, analyzed and interpreted data, drafted manuscript. DR: carried out the bioinformatic and statistic analysis, participated in study design, analyzed and interpreted data. DA: participated in the bioinformatic and statistic analysis, analyzed and interpreted data. ZBY: initiated and participated in the molecular genetic studies. UM: participated in the bioinformatic and statistic analysis. AJRH: supported bioinformatic analysis. FW: conceived the study, participated in its design and coordination, interpreted data, helped to write the manuscript. All authors read and approved the final manuscript.

## Supplementary Material

Additional file 1 **Total LTβR transcriptome in wt cells**. List of the 528 genes that were LTβR responsive in wt cells (10 h), regardless whether they were also regulated in *relA*^-/- ^or *relB*^-/- ^cells, or not. Click here for file

Additional file 2 **LTβR-responsive genes in wt cells**. List of genes that were significantly regulated in wt cells, but not in *relA*^-/- ^or *relB*^-/- ^cells (10 h; upregulation, cat I/1, n = 161; downregulation, cat I/2, n = 205). Click here for file

Additional file 3 **LTβR-responsive genes in wt and *relA*^-/- ^cells**. List of genes that were significantly regulated in wt and in *relA*^-/- ^cells (10 h; upregulation, cat II/1, n = 13; downregulation, cat II/2, n = 17). Click here for file

Additional file 4 **LTβR-responsive genes in wt and *relB*^-/- ^cells**. List of genes that were significantly regulated in wt and in *relB*^-/- ^cells (10 h; upregulation, cat III/1, n = 54; downregulation, cat III/2, n = 43; cat III/3, n = 3; cat III/4, n = 2). Click here for file

Additional file 5 **LTβR-responsive genes in wt, *relA*^-/- ^and *relB*^-/- ^cells**. List of genes that were significantly regulated in each of the genotypes (10 h; upregulation, cat IV/1, n = 20; downregulation, cat IV/2, n = 10). Click here for file

Additional file 6 **Zoomable/enlarged version of fold change heatmaps**. Heatmaps displaying the fold change values observed in the three different cell lines at 10 h compared to 0 h. For figure legend see Figure 3. Gene symbols and GenBank Accession Numbers (in brackets) are also displayed. Click here for file
