# Supplementary figures and images for: Differential RelA- and RelB-dependent gene transcription in LTβR-stimulated mouse embryonic fibroblasts
Source: BMC Genomics. 2008 Dec 16;9:606. doi: 10.1186/1471-2164-9-606 (PMC2637282; doi:10.1186/1471-2164-9-606)

Color Key

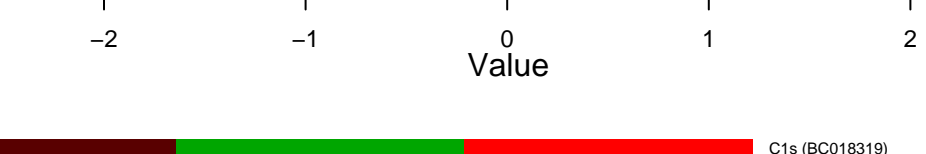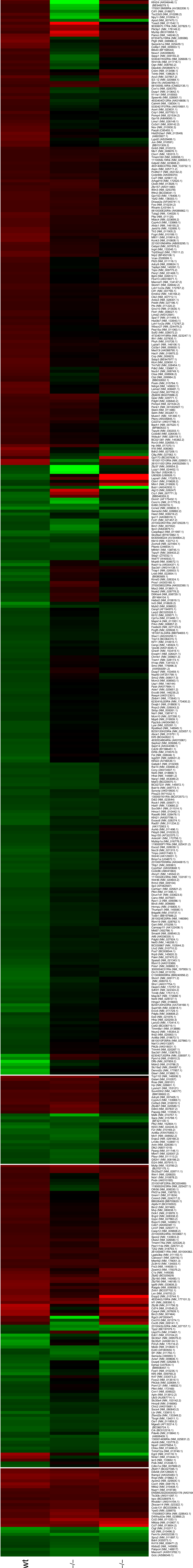

wt

relA<sup>-/-</sup>

relB<sup>-/-</sup>

Supplement: Additional file 6 — Zoomable/enlarged version of fold change heatmaps. Heatmaps displaying the fold change values observed in the three different cell lines at 10 h compared to 0 h. For figure legend see Figure 3. Gene symbols and GenBank Accession Numbers (in brackets) are also displayed. [file 1471-2164-9-606-S6.pdf]
